# Liver-resident memory T cells: life in lockdown

**DOI:** 10.1007/s00281-022-00932-w

**Published:** 2022-04-28

**Authors:** Laura J. Pallett, Mala K. Maini

**Affiliations:** Institute of Immunity & Transplantation, Division of Infection & Immunity, UCL, Pears Building, Rowland Hill St, London, NW3 2PP UK

**Keywords:** Tissue-resident, Liver, Adaptive immunity, Metabolic adaptation, CD8^+^ T_RM_, CD4^+^ T_RM_

## Abstract

A subset of memory T cells has been identified in the liver with a tissue-resident profile and the capacity for long-term ‘lockdown’. Here we review how they are retained in, and adapted to, the hepatic microenvironment, including its unique anatomical features and metabolic challenges. We describe potential interactions with other local cell types and the need for a better understanding of this complex bidirectional crosstalk. Pathogen or tumour antigen-specific tissue-resident memory T cells (T_RM_) can provide rapid frontline immune surveillance; we review the evidence for this in hepatotropic infections of major worldwide importance like hepatitis B and malaria and in liver cancers like hepatocellular carcinoma. Conversely, T_RM_ can be triggered by pro-inflammatory and metabolic signals to mediate bystander tissue damage, with an emerging role in a number of liver pathologies. We discuss the need for liver sampling to gain a window into these compartmentalised T cells, allowing more accurate disease monitoring and future locally targeted immunotherapies.

## Introduction: in lockdown or not: does it matter to liver T cells?

The liver is well-recognised to be both the central metabolic hub within the body and a complex immunological organ. In addition to nutrient storage and detoxification, its anatomical and physiological features shape an array of immune cell populations and functions that are highly specialised to this organ [1–4]. Bombarded with food and bacterial antigens from the portal vein, the liver has homeostatic tolerogenic properties, manifested in the low level of immunosuppression required to transplant HLA-mismatched livers [5, 6]. Within the immune landscape characteristic of the liver, there are some cell types that are passing through or preferentially enriched there and others that are exclusively ‘resident’. Some ‘resident’ non-parenchymal cell types comprise distinct lineages such as hepatic stellate cells or Kupffer cells (KC), whereas others are simply a subset of circulating populations that infiltrate and acquire residence in the liver. In this review, we will focus on the subset of classical αβ CD4^+^ and CD8^+^ T cells that exhibit features of ‘tissue residency’, by which we mean they infiltrate the liver and receive signals re-programming them to undergo ‘lockdown’, confined there for prolonged periods of time. Tissue-resident memory T cells (T_RM_) are characterised by distinct transcriptional, phenotypic and functional features, including long-term persistence to provide frontline immune surveillance in non-lymphoid tissues [7–11]. We will consider how liver T cell residency may occur, what contribution it makes to protective and pathological hepatic immune responses and what the clinical implications are.

The liver is a highly vascular organ, with the entire body’s blood volume passing through it every few minutes [12]. In addition to the usual arterial blood supplying all organs, the liver receives 80% of its allocation as low-pressure deoxygenated portal venous blood, draining from the gut and spleen [12]. Blood permeates the liver through narrow-lumen sinusoids, with fenestra in their endothelium providing gaps through which pseudopodia of intravascular T cells can probe for antigen presented by hepatocytes without transendothelial migration into the parenchyma [13–15]. One might therefore question whether these unique anatomical features of the liver facilitate adequate surveillance for pathogens and tissue damage by recirculating memory T cells without the need for classical tissue-residence. T_RM_ in other organs are typically extravascular, stationed in close proximity to epithelial cells in order to sensitively monitor their health and respond rapidly with frontline immunosurveillance [8, 16]. In the liver, the capacity of T_RM_ to be retained at the precise site of previous antigen encounter could allow them to provide sustained local intravascular immunosurveillance, without the need to keep hunting for their cognate antigen across the whole extensive maze-like network of the sinusoidal vasculature. T_RM_ are usually assumed to have been seeded in a non-lymphoid organ following classical priming in lymphoid tissue [17]; however the liver has the unusual capacity to prime T cells in situ [18–20], a feature which is likely to promote local development of T_RM_. Prolonged retention within the liver should also allow for selection of T_RM_ that have become particularly well-adapted to the demands of this tolerogenic niche, as discussed further below. Studying the fraction of T cells confined within the liver, as well as the recirculating fraction, will therefore allow dissection of their contribution to liver diseases, and provide vital insights into how to selectively target intrahepatic immune responses.

## How are intrahepatic T cells retained in lockdown?

One of the methods used for identification of T_RM_ in animal models relies on a fluorochrome-labelled antibody given by intravascular injection rapidly marking all cells in, or accessible to, the vasculature, and not those ‘hiding’ from labelling within the tissue parenchyma [21]. However the majority of murine hepatic T_RM_ are labelled by this approach [21, 22], either because they are intravascular or because the dye can permeate into the parenchyma through the sinusoidal fenestra. The combination of parabiosis and intravital imaging confirmed that intrahepatic T_RM_ are motile cells able to crawl along sinusoidal vessels patrolling for antigen [13] as previously described for CD8^+^ effector memory T cells (T_EM_) [14]. Intravital imaging of the non-inflamed murine liver uncovered a role for CD44 expression and its interaction with endothelium-bound platelets in promoting the initial ‘trapping’ of CD8^+^ T cells locally, rather than traditional selectin-mediated rolling interactions classically associated with T cell migration into and through other tissues [14]. The retention of murine T_RM_ within sinusoids during parabiosis experiments [13, 21] raises the question of whether this is achieved by molecular interactions occurring through fenestra with underlying local epithelia and/or stromal cells or whether liver sinusoidal endothelial cells (LSECs) themselves can tether T_RM_. One example is the expression of CD11a (ItgβL; α subunit of LFA-1), potentially interacting with ICAM-1 expressed on the surface of LSECs as well as hepatocytes, shown to be critical for both the retention and patrolling behaviour of CD8^+^ T_RM_ [23]. This is in line with the role for the more generalised LFA-1-dependent intrahepatic retention of antigen-specific T cells previously proposed [24], and the expression of Itgβ2, or CD18, the β2 subunit of LFA-1 on gut-derived T_RM_ in humans [25].

**Fig. 1:**
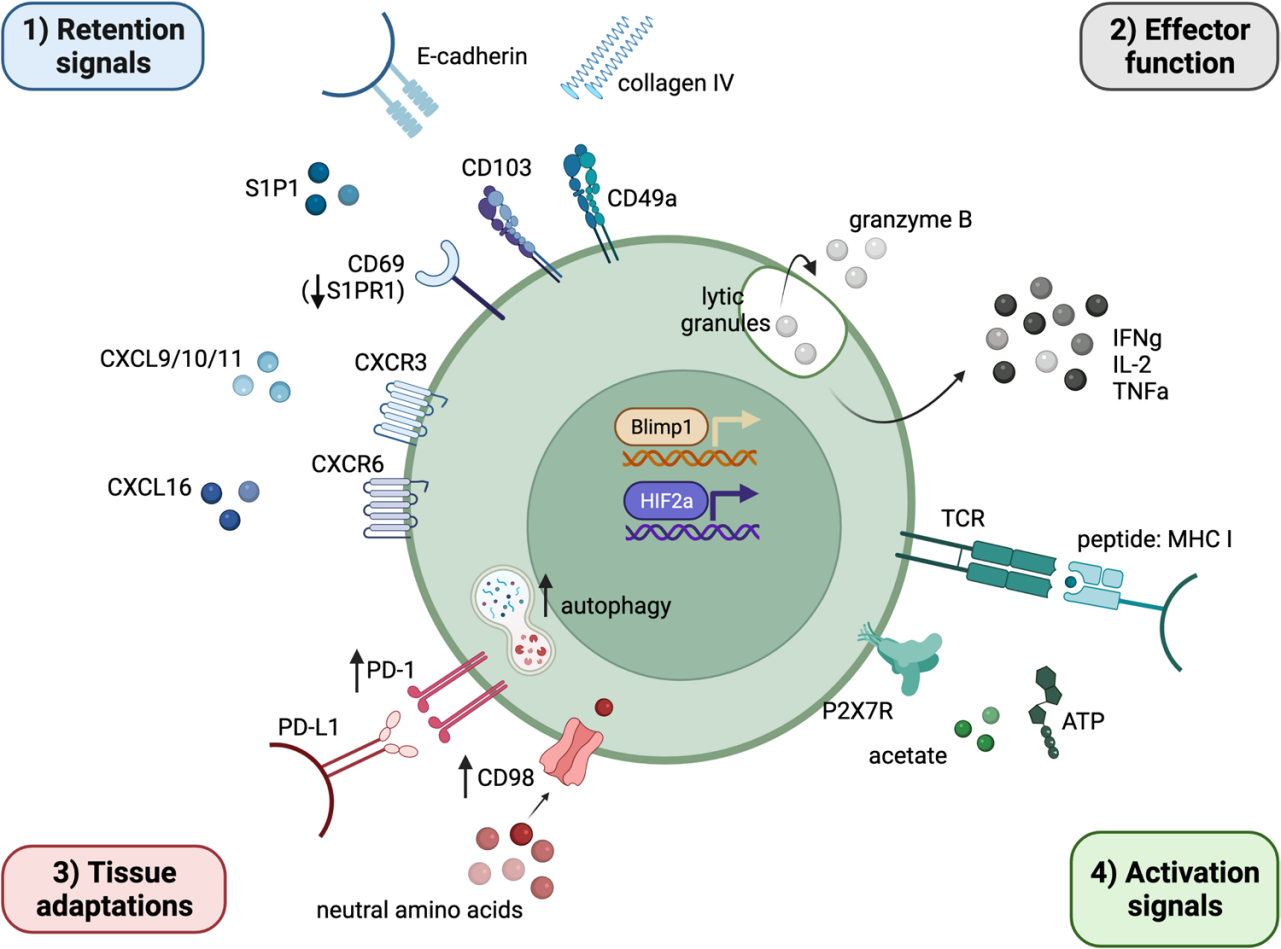
Phenotypic and functional features of human liver-resident CD8^+^ T_RM_

To define T_RM_ in humans the field has largely relied on the finding that responses with prototypic [26] surface markers, transcription factors and functional features are compartmentalised within the liver and excluded from blood [27–30]. Liver T_RM_ are transcriptionally distinct between species; murine liver CD8^+^ T_RM_ express high levels of both transcription factor homolog of Blimp-1 in T cell (Hobit) and B-lymphocyte-induced maturation protein-1 (Blimp-1), whereas human liver CD8^+^ T_RM_ cells lack Hobit, instead favouring expression of a Hobit^lo^Blimp-1^hi^Tbet^lo^ transcriptional profile (Fig. 1) [27, 28], similar to human T_RM_ found in many other tissues [26]. The C-type lectin CD69 is a functionally relevant marker for distinguishing both CD4^+^ and CD8^+^ T_RM_ from recirculating T_EM_, due to its ability to antagonise the sphingosine 1-phosphate receptor (S1PR1) that binds S1P to drive cellular egress via the lymphatics [26, 31] (Fig. 1). However, there is a growing appreciation that expression of CD69 alone is not definitive [21, 32] and that its forced expression on murine T cells confers a limited ability to generate genuine T_RM_ in vivo [33]. Recently, downregulation of S1PR5 was also shown to contribute to bidirectional tissue trafficking and retention of CD8^+^ T_RM,_ including in the murine liver [34]. Consequently, more comprehensive T_RM_ identification in the liver will require a panel of further surface markers and the use of transcriptional profiling. Notable species- and tissue-specific differences exist, adding complexity to the translation of mouse to human studies. For example, the αE integrin, CD103 is co-expressed by the majority of gut T_RM_ [21] but not on murine liver T_RM_ [13, 23, 35, 36], and only by approximately 10% of memory CD8^+^ T cells in the human liver [27] (Fig. 1).

The numerical dominance of CD69^+^ CD8^+^ T cells lacking CD103 in the human liver has led to a quest for further markers to better define intrahepatic T_RM_ and their mechanisms of retention. It is tempting to speculate that T_RM_ lacking CD103 have yet to experience prolonged exposure to environmental signals required to promote their full differentiation into CD69^+^CD103^+^, such as TGFβ or interaction with E-cadherin-expressing hepatocytes or local stroma; however, recent transcriptional profiling of such cells from multiple human tissues has confirmed this population to contain *bona fide* T_RM_ [26]. Additional markers being used in both mouse and human to define liver T_RM_ are the chemokine receptors CXCR6 and CXCR3. Supporting the role for CXCR6 and its interaction with KC-produced CXCL16, its knockdown in T cells prevents the development and maintenance of protective memory CD8^+^ T cells in the liver [37]. Similarly, production of the inflammatory chemokines CXCL9/10/11 by hepatocytes has long been associated with the migration and retention of CD8^+^ T cells in the liver [38], with the blockade of CXCR3 preventing the accumulation of T_RM_ in the murine liver [39]. Notably, both CXCR6 and CXCR3 are expressed at high levels on both *bona fide* CD69^+^CD103^+^ T_RM_ and CD69^+^CD103^−^ T_RM_-like cells in the human liver [27–29] (Fig. 1).

There is also a growing appreciation from studies in mice and/or other peripheral human tissues that integrins binding to extracellular matrix proteins promote T_RM_ retention. One such integrin is CD49a (α1β1 integrin; CD29). In the lung for example, CD49a is essential for the motility and survival of T_RM_, in part through engagement within its ligand collagen-IV directly limiting apoptosis [40, 41], whilst in the skin, CD49a marks a functionally distinct, cytotoxic subset capable of contributing to immunopathology [42]. Although limited analysis of intrahepatic T_RM_ for CD49a expression has been done, it has been observed on CD69^+^CD8^+^ T_RM_ ‘washed’ out during the perfusion of live donor organs [29]. Given the widespread expression of collagen-IV in the healthy liver [43, 44], CD49a is likely to represent a key intrahepatic T_RM_ marker, at least in the absence of advanced fibrosis, where collagen-I-III start to dominate [43, 44].

Much of what we know about liver-resident T_RM_ relates to the CD8^+^ fraction. Until recently, little work had been done to explore the hepatic compartmentalisation of CD4^+^ T cells. The enrichment of CD8^+^ T cells in the liver and lack of optimal CD4^+^ T cell priming have long been recognised [45], but whether a CD4^+^ T_RM_ fraction existed in human liver had not been investigated. Recently, we provided the first tissue residency analysis of the CD4^+^ compartment in the human liver, showing that, although they do not express CD103 (analogous to CD4^+^ T_RM_ in many other tissues) [46, 47], CD69 alone could delineate three phenotypically and functionally distinct subsets – CD69^NEG^, CD69^INT^ and CD69^HI^. Mismatched allograft data confirmed that the CD69^HI^ subset represents the long-lived, *bona fide* resident population co-expressing high levels of CXCR6, CD49a and lacking S1PR1 [47]. Instead, intermediate CD69 expression delineated a unique population, that although enriched in the liver, retained expression of chemokine receptors like CX_3_CR1 providing the potential for recirculation [47].

Moving beyond the expression of surface markers and exclusion from peripheral blood, approaches harnessing transplant tissue have enabled better interrogation of the true potential for residency in humans. Such models have enhanced our understanding of the retention, longevity and the potential for egress or replenishment of human liver-resident T_RM_. The use of HLA-specific monoclonal antibodies has allowed for the discrimination of liver-resident (donor-derived) and liver infiltrating (recipient-derived) leukocytes in explanted allografts, revealing long-lived CD4^+^ and CD8^+^ T cell progeny maintained within the liver for up to a decade after transplantation into HLA-mismatched individuals [47, 48]. Intriguingly, such an approach also demonstrated the potential for recipient-derived infiltrating T cells to acquire a tissue-residency phenotype once within the liver, thereby contributing to the local pool of resident T cells [47, 48]. Interestingly, infiltrating lymphocytes trapped within the transplanted liver equilibrated to reflect the characteristic global cellular composition of the normal liver, reinforcing the role for environmental cues in shaping the local immune cell landscape [48]. The study of material harvested post transplantation has been used to investigate T_RM_ heterogeneity in the human gut [25, 49, 50], with advanced techniques such as single-cell transcriptomics revealing the existence of CD4^+^ and CD8^+^ T_RM_ populations distinguished by their expression of either CD103 or the β2-integrin, CD18 [25]. Such unbiased analysis should likewise be used to interrogate the heterogeneity of liver T_RM_; CD18 may well be an alternative surface marker to complement CD69 when delineating the CD69^+^CD103^−^ T_RM_ pool in the liver.

Another remaining question is whether intrahepatic CD8^+^ T_RM_ ever leave the liver to migrate to local lymph nodes, or in some cases even re-enter the systemic circulation (where they may be difficult to detect if they have downregulated their characteristic retention markers). The inability of CD69^+^CD103^+^-expressing CD8^+^ T_RM_ to exit the liver or intestine via the vasculature was supported by their lack of detection in blood samples obtained directly from the hepatic and portal veins respectively [48]. However, intrahepatic CD8^+^ T_RM_ could also leave via the lymphatics; they have been observed in liver-draining lymph nodes but they lacked expression of CXCR6 so may represent a discreet population (as described in other human lymph nodes [46]) rather than ‘ex-liver T_RM_’ [48]. Upon antigenic re-exposure, murine CD8^+^ T_RM_ have been shown to be able to leave lockdown and reposition themselves in local draining lymph nodes, to supplement regionalised immunosurveillance by a process termed ‘retrograde migration’ [32, 51]. A fraction of murine T_RM_ have also recently been shown to have the capacity to re-join the circulating pool and contribute to systemic secondary responses, whilst maintaining a high propensity to home back to their tissue of origin on re-activation [52, 53]. Human skin xenograft models also showed that a fraction of CD4^+^ T_RM_ have the capacity to migrate to distant skin sites via the lymphatics and circulation [54]. Detailed TCR clonotypic and/or epigenetic profiling would be required to investigate whether an analogous population of liver T_RM_ can egress when re-stimulated with antigen, supplementing immunity in draining lymph nodes or temporarily re-joining the circulatory pool to then re-seed other parts of the liver.

## Lack of social isolation in liver lockdown

Whilst under lockdown within the liver, T_RM_ are far from socially isolated, instead being surrounded by a rich network of cells and stroma with which they can interact. Although some studies have addressed the influence of liver parenchymal and non-parenchymal cells on global intrahepatic CD4^+^ and CD8^+^ T cells, many of these have not been specifically dissected for their impact on the long-term resident fraction. Because they are confined together for prolonged periods, T_RM_ would be expected to be more heavily affected by the habits of their ‘housemates’ than immune populations that are re-circulating. Not only will other populations locked down in the liver shape hepatic T_RM_, but cellular crosstalk is likely bidirectional; T_RM_ cells would be expected to have more profound influences on other local populations than T cells that are just transiently passing through.

The narrow lumen of liver sinusoids means that incoming cells are pushed into close contact with other cells within the vasculature, as well as potentially contacting cells within the liver parenchyma through the unique liver sinusoidal endothelial fenestration. The typical model for the derivation of T_RM_ in other organs is that T cells primed in lymphoid tissue infiltrate the non-lymphoid site during the clonal expansion phase of an immune response, undergoing transcriptional reprogramming in response to antigen and/or other microenvironmental signals, and remaining ‘parked’ there [10, 55, 56]; this provides a mechanism to bias T cell specificities at tissue sites most vulnerable to re-infection or repetitive injury. Accumulating evidence suggests a pool of T cells imprinted to become T_RM_ can already be distinguished following priming by particular DC subsets within lymphoid tissue [55], but whether non-lymphoid APC can prime T_RM_ has not been resolved. The subsequent recognition of cognate antigen within the non-lymphoid organ where it takes up residence is not essential for all T_RM_ formation but can certainly promote it [57, 58]. In support of this, T cells specific for hepatotropic viruses are more likely to acquire a full residency phenotype in the human liver (CD69^+^CD103^+^) than those for non-hepatotropic viruses such as influenza-A, respiratory syncytial virus (RSV) and Epstein-Barr virus (EBV) [29, 48].

However, the liver is unusual in being a non-lymphoid organ where priming of naïve T cells is well-reported to occur; this could therefore constitute a source of hepatic T_RM_. The liver has been recognised for a number of years to house several cell types with the capacity to act as antigen presenting cells, able to prime de novo T cell responses in situ, including liver-resident DCs, LSECs, KCs and hepatocytes [18–20]. Early experiments by Bertolino and colleagues [59–61], later confirmed by others, demonstrated that when antigen expression is limited to hepatocytes, naïve T cells undergo initial activation and proliferation but fail to differentiate into functional T cells [62–64]. LSECs were similarly able to prime T cells but again in a tolerising manner [65, 66]. Instead, KC priming could induce functional CD4^+^ and CD8^+^ T cell responses [62, 67, 68]. Whereas hepatocyte priming induced loose intravascular clusters of motile cells, KC priming drove differentiation of dense clusters of extravascular immotile CD8^+^ T cells. Whilst some liver T_RM_ clearly patrol within the sinusoids [13, 21, 23, 27, 29], further studies are needed to investigate whether others accumulate in the extravascular space, perhaps as a result of KC priming.

Although liver T_RM_ express high levels of PD-1 (Fig. 1), they are not classically tolerised, being capable of rapid and protective effector function, as described for other CD8^+^ T_RM_ [26]. This conundrum suggests that either T_RM_ are in fact primed extrahepatically or that they have been ‘rescued’ from tolerance following intrahepatic priming. Such rescue of T cell effector function has been shown to be mediated by IL-2 following LSEC priming [66] and by IL-2 dependent cross-presentation by a specialised subset of KCs, denoted KC2 [69]. We have found that a hallmark of liver T_RM_ is their high IL-2 production, suggesting they, along with CD4^+^ T cells, may be able to recruit neighbouring T cells by rescuing them from tolerance. On the other hand, T_RM_-derived IL-2 could potentially also drive the development of T_REG_ [70]. More work is therefore needed to tease out whether intrahepatic priming drives liver T_RM_ localised at different sites within the liver, and whether these populations can then recruit more T_EM_ and/or T_REG_ into the liver.

Beyond antigen presentation, there are many other cellular interactions to consider within the crowded liver niche, some of which will be covered in subsequent sections on adaptations and roles of T_RM_. The liver is enriched in many innate cell types, important in their own right and for their potential to crosstalk with T_RM_. For example, we and others have defined human liver-resident NK cells and shown that they can upregulate TRAIL and NKG2D in chronic hepatitis B virus (HBV) infection and delete HBV-specific T cells [71–74]. Our new data show that intrahepatic CD8^+^ T_RM_ are also susceptible to homeostatic down-regulation by liver-resident NK cells in the setting of therapeutic vaccination in a mouse model of chronic HBV infection (CHB) (Diniz et al. in press).

## How do T cells adapt to lockdown in the liver?

When locked down in an enclosed space for prolonged periods, the availability of basic resources like nutrients can become scarce and necessitate survival adaptations. This is manifested by specific adaptations of liver T_RM_ to the hostile intrahepatic environment. Although highly vascular, the liver is hypoxic because of the deoxygenated blood it receives in the portal vein, with a zonation effect whereby oxygen tensions are particularly reduced around the central veins [75]. The cellular response to low oxygen concentrations is orchestrated by hypoxia-inducible factor (HIF1α), which can trigger the transcription of genes promoting glycolytic machinery and enhance glucose uptake through upregulation of transporters like Glut-1 [76]. Consistent with this, we noted that human liver CD8^+^ T cells expressed increased Glut-1, allowing for the enhanced uptake of glucose, in comparison to peripheral CD8^+^ T cells; this could be recapitulated by exposure of peripheral T cells to hypoxic conditions in vitro [77]. The subunit HIF2α has been found to be upregulated on human liver sinusoidal CD69^+^CD103^−^ CD8^+^ T cells [29] and tightly linked to their effector function and survival [29].

We observed that intrahepatic T cells tend to have a high proportion of depolarised mitochondria, potentially analogous to findings in intestinal intraepithelial T cells that maintain a controlled activation state despite mitochondria with reduced membrane potential [78]. Alternatively, liver-infiltrating T cells may tend to develop defective mitochondria as a result of the hostile, hypoxic microenvironment, whereas the T_RM_ fraction was noted to be relatively spared [79]. This was attributed to the amplified levels of basal autophagy we found to be a hallmark of T_RM_ (Fig. 1) providing a mechanism to recycle defective organelles (e.g. mitochondria by mitophagy) and remove excess ROS; accordingly, autophagy inhibitors recapitulated high CD8^+^ T cell mitochondrial depolarisation. Autophagy also provides biomolecules for cellular metabolism by catabolism of proteins and lipids, thus likely constitutes an important reserve supply to fuel the high functionality and longevity of T_RM_. We discovered this high level of autophagy could be imprinted on T cells by hepatic stellate cells, in an IL-15-dependent manner. IL-15 provided a mechanistic link between autophagic flux within a T cell and its ability to acquire a programme of tissue-residency, such that the in vitro derivation of T_RM_-like cells using cytokines was abrogated if autophagic flux was blocked [79].

A further metabolic adaptation we have described in human liver T_RM_ is the induction of system l-amino acid transporters, marked by the expression of CD98 (Fig. 1) [27]; these have been shown to be required for the uptake of neutral amino acids like leucine in the metabolic reprogramming underpinning the proliferative response to T cell receptor (TCR)-mediated signalling [80]. We discovered that system l-amino acid transporters could be induced by a deprivation of arginine in the T cell milieu, such as they may encounter in the liver due to arginase-producing cells and competition for arginine [81]. Granulocytic myeloid-derived suppressor cells, that accumulate in the HBV-infected liver, express high levels of arginase-I [81] and damaged hepatocytes are another source of this enzyme responsible for catabolising arginine [82, 83]. T cells need to take up large quantities of arginine for successful metabolic reprogramming [84] and hepatocellular carcinoma (HCC) can further exacerbate the competition since it is also auxotrophic for this amino acid [85].

The survival of skin T_RM_ has been shown to depend on the exogenous uptake of fatty acids, through the transporters FABP4/5, for their oxidative metabolism rather than the usual oxidation of endogenous fatty acids [86]. Recently, murine liver T_RM_ have been shown to exhibit a differential spectrum of FABPs, expressing high levels of FABP1 and some FABP4, without detectable levels of FABP5. Expression of FABP isoforms may not only be a requirement for the establishment of residency [86, 87], but may also be involved in conferring tissue specificity; intriguingly, CD8^+^ T_RM_ adoptively transferred from liver to skin adapt by increasing their expression of FABP5 upon entering their new tissue niche [87]. This differential expression pattern of FABP isoforms, and dependence on exogenous FAO, has yet to be confirmed for human liver T_RM_.

Whilst liver T_RM_ may struggle to obtain sufficient supplies of oxygen and some nutrients, they should be bathed in an excess of cholesterol [88], which has been reported to contribute to the upregulation of PD-1 in the cholesterol-rich tumour niche [89]. We postulated that exposure to a high cholesterol milieu explained our observation that T cells from the liver responded better than those from the circulation to acyl-CoA:cholersterol acyltransferase (ACAT) inhibitors that block the build-up of excess cholesterol as neutral lipid droplets and divert it to the T cell membrane to promote efficient immune synapse signalling [90]. ACAT inhibition consistently increased the functionality of HBV-specific T_RM_ extracted from human liver samples, exemplifying the potential to target metabolic checkpoints as novel immunotherapies [90].

Beyond metabolic features, the regulation of liver T_RM_ to maintain a homeostatic state of tolerance, yet remain poised for rapid immune surveillance, is a key liver adaptation. The PD-1 axis is central to liver tolerance [19], with parenchymal and non-parenchymal cell types expressing PD-L1 in the liver. T_RM_ are adapted to the liver by expressing high levels of the immune checkpoint molecule PD-1, yet do not demonstrate functional features of T cell ‘exhaustion’. Paradoxically, PD-1^hi^ liver-resident CD8^+^ T_RM_ remain functionally superior to non-resident T cells, with rapid cytokine production upon TCR engagement (Fig. 1) [27, 29]. Although apparently at odds with the vigilance required by T_RM_, such high levels of PD-1 may impose some level of restraint on local effectors, preventing unnecessary immune damage upon repetitive stimulation. This functional relevance of PD-1 expression on T_RM_ was corroborated by a recent study revealing PD-1^hi^ human pancreatic T_RM_ could be regulated by PD-L1^+^-tissue macrophages [91]; therefore, it is highly likely that liver T_RM_, resident in the sinusoids, are functionally regulated by PD-L1-expressing cells in the liver to restrain them in the homeostatic state of tolerance characteristic of the liver. As discussed above, their high IL-2 production likely allows liver T_RM_ to overcome PD-1-mediated tolerance upon antigen encounter. The functional relevance of cell-intrinsic IL-2 production by CD8^+^ T cells has been clarified by a recent study showing that it limits their capacity to receive IL-2-dependent Stat-5 signals, thereby promoting stem-like survival and resistance to exhaustion, resulting in more effective viral control [92].

## Protective potential of liver TRM

T_RM_ are the frontline of our adaptive cellular defence in many peripheral tissues including the liver. T_RM_ can provide rapid and potent protection against a diverse range of bacterial, viral and parasitic infections, associate with improved tumour control and prognosis and have an emerging potential to also regulate tissue damage and fibrosis [7, 9, 39, 93–98].

The first definitive demonstration of a protective advantage from the hepatic CD8^+^ T_RM_ population came from a series of important studies showing their critical role against malaria liver-stage infection [13, 37, 57, 99–102]. Previous studies had suggested intrahepatic populations of IFNγ-producing cells were more efficient at providing the very large numbers of memory T cells required for malaria protection [103, 104]. Parabiosis experiments then identified T_RM_ forming in the liver after different malaria vaccination strategies, depletion of which ablated protection from infection [13, 105]. The longevity of hepatic T_RM_ was postulated to be a key correlate of protection in subjects receiving a liver-targeted *Plasmodium falciparum* (Pf) sporozoite vaccine; challenge experiments at 59 weeks showed protection against parasitaemia outlasted the waning of antibodies and circulating Pf-specific T cells [101]. A role for long-lived hepatic T_RM_ in this setting was supported by vaccination of non-human primates, showing Pf-specific T cells were enriched within the liver by ~ 100-fold compared to the periphery [101]. As discussed in the final section, these studies have informed malaria vaccine strategies tailored to promoting the induction of liver T_RM_.

Our studies provided the first characterisation of virus-specific CD8^+^ T_RM_ in the human liver and demonstrated that these could be long-lived [27, 48, 106]. Higher frequencies of liver CD8^+^ T_RM_ associated with well-controlled infection [27], extending previous data showing an enrichment of HBV-specific CD8^+^ T cells in the liver of subjects with low viral load [107, 108]. Intrahepatic CD8^+^ T cells with a T_RM_ phenotype were directed against all major HBV proteins and persisted in the liver following resolution of infection [27]. Further work is needed to understand to what extent circulating HBV-specific T cell responses simply under-represent the magnitude and potency of intrahepatic responses or whether particular specificities may end up completely compartmentalised within the liver of some subjects such that the blood does not always represent the full breadth of viral regions targeted. Certainly, having HBV-specific liver T_RM_ stationed within the liver with the capacity for rapid and protracted antiviral effector function upon HBV recrudescence is a useful therapeutic goal for this hepatotropic viral infection. Antigen-specific liver T_RM_ act as an immediate first line of defence, potently producing antiviral cytokines, such as IFNγ and TNF upon TCR engagement by their cognate antigen (Fig. 1) [27, 29]. The rapidity with which T_RM_ can produce cytokines like IFNγ has been suggested to result from their increased storage of deployment-ready mRNA for these antiviral mediators [109]. Such mediators inhibit HBV replication in a non-cytopathic manner [110, 111] and activate the secretion of chemokines by parenchymal and non-parenchymal cells involved in the recruitment of non-antigen specific cells [112]. Liver T_RM_ cells themselves are also capable of producing and secreting large amounts of such chemokines, for example MIP1β, that should enhance recruitment of inflammatory, non-antigen specific effectors. Intriguingly antigen-specific and bystander liver CD8^+^ T_RM_ also produce high levels of IL-2 extremely rapidly [27, 29]; such autocrine IL-2 promotes the expansion of a memory CD8^+^ T cell pool [113] and may help to combat T cell apoptosis and liver antigen-presenting cell-induced CD8^+^ T cell dysfunction, as discussed earlier [62, 69]. Our study and one in hepatitis C virus (HCV)-infected livers [28] showed human liver CD8^+^ T_RM_ have reduced ex vivo granzyme B and perforin (Fig. 1), which may help to limit damage to this vital organ in the steady state. However, in the context of other studies discussed, liver T_RM_ can become cytotoxic killers able to eliminate malaria-infected cells or drive immunopathology upon exposure to particular stimuli.

Beyond their roles in pathogen defence, CD8^+^ T_RM_ have been shown to be critical for anti-tumour control in murine models, with accumulating data supporting a protective role in various human cancers [7, 94, 97]. Several recent studies have addressed the role for CD8^+^ T_RM_ in human HCC using combinations of high dimensional approaches for detailed dissection of the immune landscape of HBV-related and non-viral HCC in tissue samples. Using multiplexed immunofluorescence, Lim et al. found that CD8^+^ T_RM_ are not only enriched in the HBV-related HCC microenvironment, but their presence is associated with improved overall patient survival [114]. This was further investigated in an elegant study by Chen*g* et al. using highly multiplexed peptide-MHC tetramers, identifying 91 different HBV and tumour antigen-specific T cell specificities, to show that patients with higher frequencies of intratumoral antigen-specific CD8^+^ T_RM_ had a longer relapse-free survival [115].

Another previously unappreciated protective role for CD8^+^ T_RM_ has recently emerged from a study implicating CD8^+^ T_RM_ and the Fas-FasL pathway in the resolution of liver fibrosis [39]. In a mouse model of diet-induced non-alcoholic steatohepatitis (NASH, also known as metabolic liver disease), CD8^+^ T_RM_ were reported to increase in frequency in those mice resolving fibrosis, whilst their in vivo depletion prevented fibrosis resolution. Conversely, adoptive transfer of CD8^+^ T_RM_ was anti-fibrogenic, via their ability to predispose activated stellate cells (myofibroblasts) to Fas-FasL-mediated apoptosis [39]. CD8^+^ T_RM_ were noted to accumulate within fibrotic tracts of human liver but their anti-fibrogenic potential in human liver fibrosis of different aetiologies has not yet been defined. Of note, it is likely that human CD8^+^ T_RM_ would have to undergo transendothelial migration to maintain anti-fibrotic (and other protective) effects once capillarisation and defenestration develop, as these pathological sequalae of fibrosis have been shown to limit immunosurveillance of extravascular targets by intrasinusoidal T cells [14].

## Pathogenic potential of liver TRM

The dual potential of hepatic T cells to provide protection against infected cells but drive immunopathology when mis-directed against heathy tissue has long been recognised. Recently, liver T_RM_ have similarly been demonstrated to have pathogenic as well as protective potential, with the discovery of a novel mechanism of bystander (as opposed to antigen-specific) killing, triggered by a metabolic signal.

In two recent studies using pre-clinical models of NASH and NASH-related HCC [116, 117], the authors demonstrated an accumulation of activated hepatic CD8^+^ T cells with a tissue-residency phenotype (in one study: CXCR6^+^PD-1^hi^, but lacking CD49a [116]; the other CD69^+^PD-1^+^CD44^+^ [117]) that correlated with the level of immune-mediated damage, and progression to HCC. Mechanistically, Dudek et al. propose that short-lived CXCR6^hi^CD8^+^ T_RM_ promoted the non-specific killing of hepatocytes, a process termed ‘auto-aggression’. The characteristic disturbance in lipid metabolism associated with NASH contributed to the auto-aggression of IL-15-activated CD8^+^ T cells due to increased metabolic stimulation from mediators such as acetate and extracellular ATP signalling through purinergic P2RX7 receptors [116] that are known to be highly expressed by T_RM_ [118, 119] (Fig. 1). Importantly, in the first of these two studies, therapeutic blockade of the FasL pathway offered protection against auto-aggressive CD8^+^ T cells, uncovering the potential to limit liver damage in chronic liver diseases such as NASH (and therefore preventing the development of HCC), without compromising the efficacy of antigen specific immunity [116]. Instead, the study by Pfister et al. suggested the aberrantly activated, PD-1^+^CD8^+^ T_RM_ produced large amounts of TNF, that led to ineffective immunosurveillance, contributed to tumour progression, and decreased immunotherapy efficacy [117].

In support of these data in murine NASH, human liver CD8^+^ T cells characterised by high levels of CD69 expression have also been reported to promote bystander, non-antigen specific liver damage via IL-15-induced pathways; sinusoidal CD69^+^CD8^+^ T_RM_ with increased HIF2α and NKG2D expression positively correlated with the degree of liver failure and disease severity in patients with ongoing liver damage resulting in end-stage cirrhosis [29]. A new study using single cell RNA sequencing also linked hepatic CXCR6^hi^ CD8^+^ T cells with non-specific cytotoxic activity through Fas-FasL in patients with HBV experiencing hepatic flares [120]. Intrahepatic CD8^+^ with a T_RM_ phenotype have similarly been shown to increase in paediatric acute liver failure [121] and in autoimmune hepatitis [122], with numbers associating with severity in the latter study. Thus, it remains possible that more detailed characterisation will allow protective versus pathogenic subsets of liver CD8^+^ T_RM_ to be defined. Alternatively, and perhaps more likely, the same phenotypically identified populations can act as useful effectors when eliminating cells expressing their cognate viral/tumour antigen but also as mediators of non-antigen specific killing in the presence of particular signals such as the inflammatory cytokines and metabolic signals described above.

Finally, as we have previously noted in this review, limited work has been done on tissue-resident CD4^+^ T cells; however, our initial profiling revealed that one liver-enriched subset, marked by CD69^INT^ expression, exhibited pro-fibrogenic potential, producing IL-4 upon TCR engagement and correlated with the extent of necroinflammation in a small cohort of CHB patients [47]. The capacity of liver T_RM_ to sense and drive tissue damage strengthens the need for further studies into their role in regulating liver inflammation and fibrosis.

## Clinical sampling and therapeutic targeting of liver TRM

The majority of immunological studies aiming to understand liver disease pathogenesis or monitor immunotherapies have relied on peripheral blood sampling; this has allowed many informative insights and will remain the staple approach going forward. It has always been considered to be an advantage to augment such studies using samples from the site of disease whenever possible, but our increasing understanding of tissue-resident populations has further underscored this need. Growing reliance on non-invasive methods of assessing liver disease is increasingly limiting availability of liver biopsy tissue for immunology studies [123]. However, our paired comparison of the flow cytometric analysis of cellular yields from a traditional core biopsy and the much less invasive fine needle aspirate (FNA, using a 22-guage spinal needle) showed a broadly comparable capacity to sample the liver immune landscape, including resident T and NK cells [106]. Since FNA can be used for repeated longitudinal sampling [124] and require no tissue processing, they provide a compelling approach for monitoring in vivo immune responses to novel therapeutic interventions, such as new regimes aiming to achieve therapeutic cure of CHB. Whilst FNA are appealing for monitoring novel therapies, they do not provide any histology and cannot shed light on immune cell interactions. For better understanding of liver disease pathogenesis, archival tissue blocks from previous biopsies as well as from tissue resections and explants can now be studied with new multiparameter techniques including tissue mass cytometry and spatial transcriptomics; this will allow an unprecedented window into the topology of liver-resident immune cells in relation to each other and to infected/diseased parenchymal and non-parenchymal cells. Another useful development for the study of human liver T_RM_ has been the discovery that they can be isolated in large numbers from liver transplant perfusates or ‘wash-outs’ [27, 29]. This has facilitated access to large numbers of T_RM_ from relatively healthy livers for studies into their homeostatic features.

A better understanding of tissue-resident T cell immunity is also informing the development of liver-targeted immune interventions. Having shown that malaria immunity is dependent on liver-resident T cells, several laboratories are testing strategies to selectively expand these by vaccination. The liver has been shown to provide a flexible niche with space for multiple rounds of expansion of local T_RM_ [57]. Thus, vaccine delivery could aim to direct and trap intrahepatic T_EM_, having first achieved their immunogenic priming in lymphoid organs to avoid the tolerogenic properties of the liver. This is exemplified by the prime-and-trap vaccination strategy developed by the Heath laboratory to induce liver-resident T cell in malaria; following priming of *Plasmodium*-specific CD8^+^ T cells by splenic DC, they are recruited and ‘trapped’ in the liver by recognition of hepatocyte-expressed antigen encoded by an adeno-associated viral vector [13]. A more recent approach by this group utilised a self-adjuvating glycoprotein-peptide vaccination that harnesses NKT cell ‘help’ to induce the formation of liver CD8^+^ T_RM_ cells expressing canonical markers associated with residency [100]. The route of vaccine delivery is a simple way of targeting their immunogenicity to the required organ; just as some vaccines already target the gut through oral administration and lungs through nasal or aerosolised delivery [125, 126], the liver can be targeted by intravenous (rather than intramuscular) delivery of vaccines [99, 101].

Targeting T cell boosting to the liver may serve as a useful strategy to improve not only efficacy but also safety. For example, to circumvent systemic toxicity of checkpoint inhibitors, a liver-directed locked nucleic acid oligonucleotide targeting the PD-L1/PD-1 pathway is currently being trialled in CHB. This is more likely to boost endogenous T cells with antiviral efficacy since HBV-specific T cells are concentrated in the liver, whilst being less likely to cause autoimmunity at other sites if the T cells responding to PD-1 blockade remain compartmentalised within the liver. Attempts are also being made to develop effective small molecule PD-1 inhibitors; as oral agents, these would be concentrated in the gut and, via the portal circulation, the liver, and would also therefore be expected to have dominant effects on T_RM_ locally. Monitoring tissue-resident T cell immunity is particularly pertinent for these types of liver-directed immunotherapy since expansions in hepatic T_RM_ are unlikely to be reflected in the periphery; this was nicely demonstrated in the Ishizuka study, where the increased T_RM_ achieved by intravascular vaccine delivery were only detectable once liver sampling in chimpanzees was carried out [101].

The studies described above, highlighting immunopathological roles for hepatic T_RM_, point to the need to carefully consider the merits and risks of their expansion or ablation in different disease settings. Better distinguishing the features of the fraction of T cells with stable residence in the liver able to mediate pathogenic outcomes, and their specific drivers, may allow their therapeutic elimination or blockade. If the disease-mediating fraction is localised to the liver, this raises the possibility of being able to target them locally in a much more precise and safe manner than has been possible with systemic immunosuppression. Therefore, high-dimensional phenotypic studies of human intrahepatic immune responses are urgently needed in liver diseases currently lacking specific treatments, both for understanding disease pathogenesis and for predicting relapse and treatment response. Rather than giving systemic immunosuppressive drugs like corticosteroids, with all the resultant risks, the ultimate goal would be to ablate or inhibit pathogenic T cells locally at the site of disease.

## Conclusions and future directions

Studying immune responses from the site of disease has always been an important goal; our increasing understanding of the extent of tissue compartmentalisation of immunity has further emphasised the need for this. Analysing liver-resident T cells has only just started to uncover insights into the organ-specific influences they are subject to, and the protective immunosurveillance versus pathological disease-inducing roles they play. Future studies need to examine the crosstalk of T_RM_ with other resident and infiltrating immune cells, particularly using in situ analysis to examine topological relationships. Whilst liver-resident NK cells [71] and γδ T cells [127, 128] have also been recently defined, the potential for other cells such as B cells to take up liver residence remains to be investigated. The stromal network is emerging as a powerful force shaping the behaviour of tissue-resident immunity [129–131] and one that merits investigation in the liver. It will be interesting to probe the antigen specificity of liver-resident T cells, and to what extent this is reflective of local antigen priming. Although the liver is not regarded as a classical barrier organ, it is in constant contact with the gut microenvironment through its portal blood supply, necessitating studies on the influence of the microbiota and microbial products on liver T_RM_. Pathogen-specific liver T_RM_ are already being targeted therapeutically, but manipulation of non-antigen-specific T_RM_ populations needs to address the emerging delicate balance between their protective and pathogenic potential.
